# CircRNA/lncRNA–miRNA–mRNA network and gene landscape in calcific aortic valve disease

**DOI:** 10.1186/s12864-023-09441-y

**Published:** 2023-07-25

**Authors:** Yuqi Zheng, Shuyu Wen, Shijiu Jiang, Shaolin He, Weihua Qiao, Yi Liu, Wenling Yang, Jin Zhou, Boyuan Wang, Dazhu Li, Jibin Lin

**Affiliations:** 1 Department of Cardiology, Union Hospital, Tongji Medical College, Huazhong University of Science and Technology, Wuhan, 430022 China; 2 Hubei Key Laboratory of Biological Targeted Therapy, Tongji Medical College, Union Hospital, Huazhong University of Science and Technology, Wuhan, 430022 China; 3 Hubei Provincial Engineering Research Center of Immunological Diagnosis and Therapy for Cardiovascular Diseases, Union Hospital, Tongji Medical College, Huazhong University of Science and Technology, Wuhan, 430022 China; 4 Department of Cardiovascular Surgery, Union Hospital, Tongji Medical College, Huazhong University of Science and Technology, Wuhan, 430030 China; 5 Department of Cardiology, The First Affiliated Hospital, Shihezi University, Shihezi, 832000 China

**Keywords:** Calcific aortic valve disease, Competitive endogenous RNA network, Valve interstitial cell, Valvular endothelial cell, Gender, Bicuspid aortic valve, Immune infiltration

## Abstract

**Background:**

Calcific aortic valve disease (CAVD) is a common valve disease with an increasing incidence, but no effective drugs as of yet. With the development of sequencing technology, non-coding RNAs have been found to play roles in many diseases as well as CAVD, but no circRNA/lncRNA–miRNA–mRNA interaction axis has been established. Moreover, valve interstitial cells (VICs) and valvular endothelial cells (VECs) play important roles in CAVD, and CAVD differed between leaflet phenotypes and genders. This work aims to explore the mechanism of circRNA/lncRNA–miRNA–mRNA network in CAVD, and perform subgroup analysis on the important characteristics of CAVD, such as key cells, leaflet phenotypes and genders.

**Results:**

We identified 158 differentially expressed circRNAs (DEcircRNAs), 397 DElncRNAs, 45 DEmiRNAs and 167 DEmRNAs, and constructed a hsa-circ-0073813/hsa-circ-0027587–hsa-miR-525-5p–SPP1/HMOX1/CD28 network in CAVD after qRT-PCR verification. Additionally, 17 differentially expressed genes (DEGs) in VICs, 9 DEGs in VECs, 7 DEGs between different leaflet phenotypes and 24 DEGs between different genders were identified. Enrichment analysis suggested the potentially important pathways in inflammation and fibro-calcification during the pathogenesis of CAVD, and immune cell patterns in CAVD suggest that M0 macrophages and memory B cells memory were significantly increased, and many genes in immune cells were also differently expressed.

**Conclusions:**

The circRNA/lncRNA–miRNA–mRNA interaction axis constructed in this work and the DEGs identified between different characteristics of CAVD provide a direction for a deeper understanding of CAVD and provide possible diagnostic markers and treatment targets for CAVD in the future.

**Supplementary Information:**

The online version contains supplementary material available at 10.1186/s12864-023-09441-y.

## Background

Calcific aortic valve disease (CAVD) is a common valve disease, with nearly 13% people over 65 years old suffering from this disease, and a potential 2.4-fold increase expected over the next 20 years [1, 2]. Recent studies have found that CAVD is a disease involving inflammation, lipoprotein deposition and calcified nodule formations [3], but the exact pathogenesis is still unclear. Aortic valve replacement (AVR) is still the only option for patients at an advanced stage due to the lack of effective drugs. Therefore, exploring the underlying mechanisms of CAVD and searching for diagnostic markers and therapeutic drugs targets are important.

Non-coding RNAs (ncRNAs) play important roles in maintaining genome integrity, regulating gene expression and determining cell fate [4]. MicroRNAs (miRNAs), which belong to the small ncRNAs (less than or equal to 200nt) regulate gene expression by binding to the 3’UTR of the target gene [5], and have been found to play roles in CAVD. For example, miRNA-141 reduced aortic valve calcification by inhibiting the expression of bone morphogenetic protein-2 (BMP-2) [6], and miRNA-204 has been found to be a protective factor by inhibiting the calcification induction factors transforming growth factor-beta 1 (TGF-β1) and BMP2, which inhibit the transformation of valve stromal cells to osteogenesis [7]. Long non-coding RNA (lncRNA) is a kind of linear ncRNA with a length greater than 200nt, which regulates gene expression via miRNA sponging, direct binding to proteins or acting as a scaffold to recruit transcriptional factors [8]. Some lncRNAs have been confirmed to be involved in CAVD, such as lncRNA H19, which inhibited the transcription of Noch1, while Noch1 negatively regulated the progression of CAVD [9]. Another lncRNA, MALAT1, can sponge miRNA-204 and accelerate the progression of CAVD [10]. Circular RNAs (circRNAs) are formed by the covalent binding of the 3’ and 5’ ends by reverse splicing. RNA sequencing analysis showed that there were many differentially expressed circRNAs in calcified aortic valves compared to normal valves, and numerous miRNA response elements were found on these circRNAs [7]. Meanwhile, the competitive endogenous RNA (ceRNA) hypothesis suggests that circRNAs and lncRNAs can adsorb and inhibit the function of miRNAs and relieve their inhibitory effects on mRNAs [11]. Therefore, circRNAs and lncRNAs may regulate gene expression at transcriptional and post-transcriptional levels through miRNAs.

The circRNA/lncRNA–miRNA–mRNA interaction axis has been explored in many diseases, bringing new targets for diagnosis and treatment. With the development of RNA sequencing technology, many differentially expressed mRNAs, miRNAs, circRNAs and lncRNAs have been identified in CAVD patients, but no circRNA/lncRNA–miRNA–mRNA interaction axis has been established. Therefore, we use bioinformatics methods to identify differentially expressed circRNAs, lncRNAs, miRNAs and mRNAs from existing microarray and sequencing data and construct an interaction network.

The aortic valve is composed of three tissue layers: the fibrosa facing the aorta, the ventricularis facing the left ventricle outflow tract and the spongiosa [3]. As important cells in the aortic valve tissue, the phenotypic changes in valve interstitial cells (VICs) are related to CAVD [12]. Another important cell type is the valvular endothelial cells (VECs), as a barrier between the blood and interstitial tissue, which react with different shear stresses and also play a role in CAVD [13]. Moreover, the pathogenesis of CAVD is similar to that of atherosclerosis, the early stages showing obvious characteristics of inflammation, involving macrophages, T lymphocytes and mast cells [14]. Hence, although a number of differentially expressed genes (DEGs) in VECs and VICs between CAVD and normal controls have been identified [14], and several genes in VICs involved in CAVD have been found [15, 16], we applied several bioinformatics methods, including immune infiltration as well as single-cell RNA sequencing (scRNA-seq) clustering, to further explore the pathogenesis of CAVD on the VECs and VICs levels.

Bicuspid aortic valve (BAV) is a congenital aortic valve malformation and an important risk factor for CAVD. BAVs affect nearly 1.3% of the general population [17], and patients with BAVs are more likely to have VECs damage resulting from hemodynamic abnormality, followed by leaflet sclerosis and calcification [18]. However, the specific differential pathogenic mechanisms are unclear. In addition, the incidence and manifestations of CAVD vary among genders. There is evidence to suggest that the CAVD burden and aortic valve composition are different between different genders [19]. Additionally, an in vitro study found that human VICs isolated from males have a higher calcification potential than VICs from females [20]. These studies suggest that CAVD has different genetic patterns in different genders. Therefore, we further analyzed the DEGs between BAVs and tricuspid aortic valves (TAVs), as well as in different genders.

In brief, a circRNA/lncRNA–miRNA–mRNA network was constructed by comprehensive analysis of the existing public RNA datasets on CAVD. In addition, we performed subgroup analysis on the important characteristics of CAVD, such as key cells, leaflet phenotype and gender. Our results contribute to a better understanding of the pathogenesis of CAVD and provide evidence for future drug development.

## Results

### Identification of differentially expressed circRNAs (DEcircRNAs), lncRNAs (DElncRNAs), miRNAs (DEmiRNAs) and mRNAs (DEmRNAs) in CAVD

**Fig. 1:**
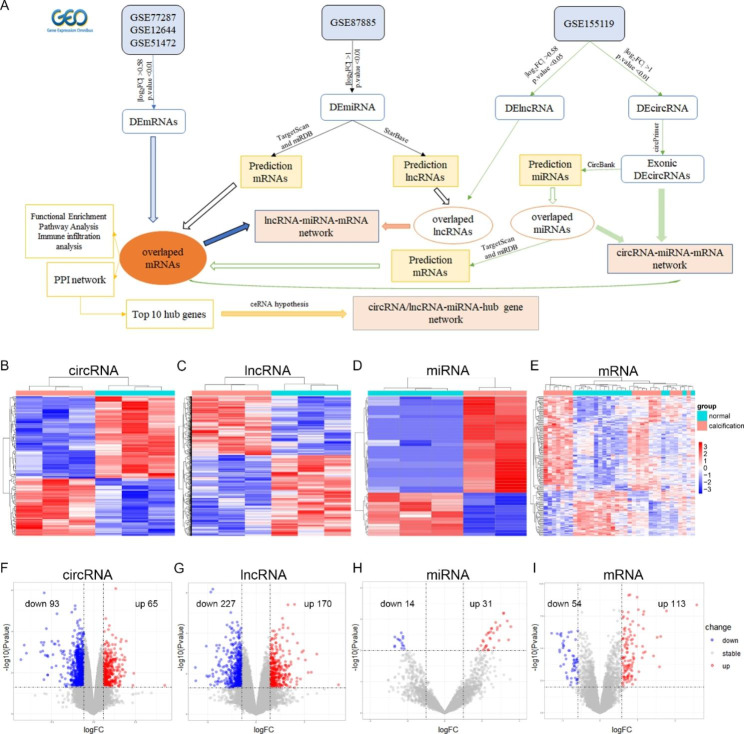
Identification of DEcircRNAs, DElncRNAs, DEmiRNAs and DEmRNAs between CAVD and the normal controls. (**A**) Workflow of bioinformatics analysis. (**B-E**) Heatmaps of DEcircRNAs, DElncRNAs, DEmiRNAs and DEmRNAs. (F-I) Volcano plots of DEcircRNAs, DElncRNAs, DEmiRNAs and DEmRNAs.

The process of differentially expressed RNA filtering is shown in Fig. 1A. After the merging and batch normalization of the downloaded datasets, principal component analysis (PCA) results show that the samples were clearly separated between the normal control group and CAVD group (Figure S1 A-C). The “limma” package of R software was used to identify DEcircRNAs, DElncRNAs, DEmiRNAs and DEmRNAs. In the GSE155119 dataset, a total of 929 DElncRNAs and 158 DEcircRNAs, including 65 that were up-regulated and 93 that were down-regulated (Table S1), were identified. The DElncRNAs were annotated by the application platform files, and 397 annotated DElncRNAs including 170 up-regulated and 227 down-regulated examples were used for further analysis (Table S2). A total of 45 DEmiRNAs were identified from the GSE87885 dataset, of which 31 were up-regulated and 14 were down-regulated (Table S3). A total of 167 DEmRNAs were identified in the merged datasets GSE77287, GSE12644 and GSE51472, of which 113 were up-regulated and 54 were down-regulated (Table S4). These DEcircRNAs, DElncRNAs, DEmiRNAs and DEmRNAs are represented by volcanic plots and heatmaps (Fig. 1B-I).

The process of the circRNA/lncRNA–miRNA–mRNA network architecture is shown in Fig. 1A. CircRNAs can be classified into three types, including exon type, intron type and exon–intron type. After excluding DEcircRNAs for which the annotation results were inconsistent with circBase, 118 exonic DEcircRNAs, 6 exon–intronic DEcircRNAs and 1 intronic DEcircRNA were obtained (Table S1). Then, miRNAs interacting with exon DEcircRNAs were predicted in CircBank, and 2231 miRNAs were predicted. Then, 36 overlapped miRNAs were obtained by overlapping the predicted results with the 45 DEmiRNAs from GSE87885 (Figure S1 D and Table S5). Next, 7856 mRNAs interacting with the 36 overlapped miRNAs were obtained from both TargetScan and miRDB. Finally, 70 overlapped mRNAs were obtained by overlapping the 7856 predicted mRNAs with the 167 DEmRNAs identified from the three datasets (GSE77287, GSE12644 and GSE51472) (Figure S1 E and Table S6).

The 45 DEmiRNAs identified from GSE87885 were used to predict the lncRNA interactions in StarBase, and only four of them had 243 interaction lncRNAs. Then, these four miRNAs predicted 1798 target mRNAs in both TargetScan and miRBD. By overlapping the 243 predicted lncRNAs and 1798 target mRNAs with the 397 annotated DElncRNAs (identified from GSE155119) and 167 DEmRNAs (identified from the 3 datasets), respectively, we obtained 3 overlapped lncRNAs and 29 mRNAs (Figure S1 F-G and Table S7).

Finally, we constructed a circRNA/lncRNA–miRNA–mRNA network containing 64 circRNAs, 3 lncRNAs, 27 miRNAs and 70 mRNAs by integrating the above interactive RNAs (Figure S2 and Table S8).

### Functional Enrichment, Pathway Analysis and protein–protein Interaction (PPI) Network Construction

The “clusterProfiler” package of R software was used for GO annotation and KEGG pathway enrichment analysis to understand the potential biological functions of DEmRNAs in the ceRNA network. The results show that the 70 DEmRNAs in the ceRNA network mainly participate in leukocyte migration, activation, chemotaxis, degranulation and regeneration coming under the term biological process (BP). The external side of the plasma membrane and the collagen-containing extracellular matrix were the main cellular components (CC). According to the molecular function (MF), these DEmRNAs were mainly enriched in terms of cytokine and chemokine activation, CXCR chemokine receptor binding, G protein-coupled receptor binding, chemokine receptor binding, and WNT protein binding (Figure S3 A). In KEGG pathway enrichment analysis, these DEmRNAs are mainly involved in the “chemokine signaling pathway”, “cytokine-cytokine receptor interaction”, “Toll-like receptor signaling pathway”, and “WNT signaling pathway” (Figure S3 B). The entries for GO and KEGG enrichment analysis are provided in Table S9.

The STRING database was used to establish the potential PPI network in CAVD. The threshold was an interaction score > 0.4, and the nodes not connected to the main network were excluded, after which a PPI network with 69 edges and 38 nodes was obtained. According to the MCC algorithm, we selected the top 10 genes in the PPI network as hub genes, including CXCL12, CCL5, CCR5, CXCL9, CD163, CD28, SPP1, HMOX1, KIT and MMP13 (Figure S3 C).

### Construction of circRNA/lncRNA–miRNA–hub gene network

**Fig. 2:**
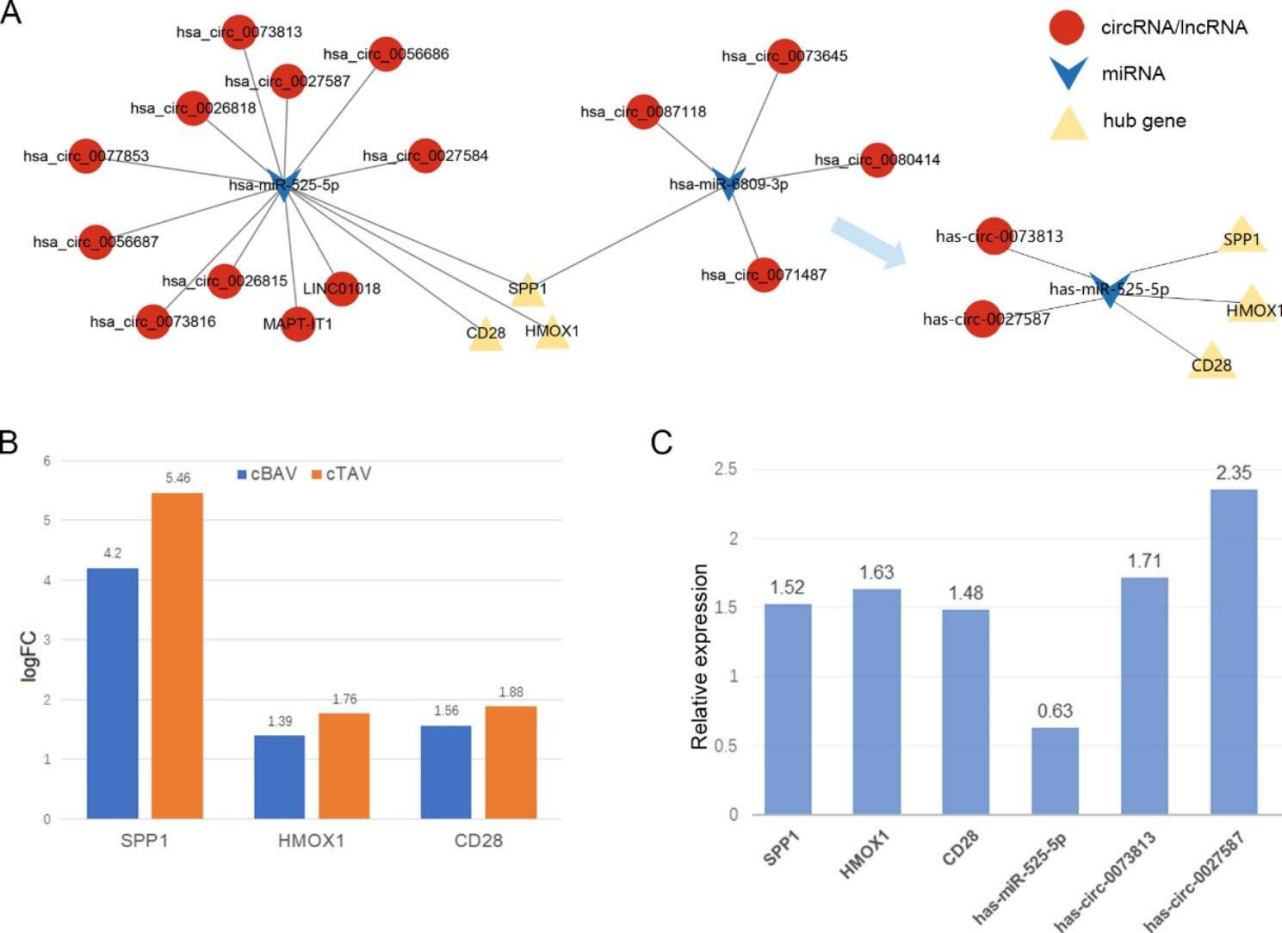
The circRNA/lncRNA–miRNA–hub gene network and validation of the hub genes, miRNAs and circRNAs. (**A**) The circRNA/lncRNA–miRNA–hub gene network was constructed according to the ceRNA hypothesis and a subnetwork confirmed by qRT-PCR. Red and yellow represent up-regulation and blue means down-regulation. (**B**) GSE148219 and GSE76718 were used to validate the hub genes, and the intensity of differential expression is shown as a bar graph. (**C**) The relative expression of hub genes, miRNAs and circRNAs by qRT-PCR. Results are expressed as fold changes of control group

According to the ceRNA hypothesis, circRNAs or lncRNAs can inhibit miRNAs, while miRNAs inhibit mRNAs. Therefore, after excluding the unmatched RNA interaction pairs, a circRNA/lncRNA–miRNA–hub gene network was constructed, containing 13 circRNAs, 2 lncRNAs, 2 miRNAs and 3 hub genes (Fig. 2A).

### Verification of hub genes, miRNAs and circRNAs in the ceRNA network

The three hub genes (SPP1, HMOX1, and CD28) in the ceRNA network obtained from our comprehensive analysis were verified in the GSE148219 and GSE76718. The results show that their expression was up-regulated either in calcified BAVs (cBAVs) or TAVs (cTAVs) compared with normal controls, which was consistent with our results (Fig. 2B).

Quantitative real-time polymerase chain reaction (qRT-PCR) was used to further verify the hub genes (SPP1, HMOX1 and CD28), miRNAs (hsa-miR-525-5p and hsa-miR-6809-3p) and the top differentially expressed circRNAs (hsa-circ-0073813, hsa-circ-0073816, hsa-circ-0027587 and hsa-circ-0073645) in the ceRNA network from clinical CAVD and non-calcific valve samples. The results show that compared with the control group, the CAVD group had a significant increase in all hub genes (SPP1, HMOX1 and CD28), hsa-miR-525-5p, hsa-circ-0073813 and hsa-circ-0027587 (Fig. 2C). Finally, a hsa-circ-0073813/hsa-circ-0027587–hsa-miR-525-5p–SPP1/HMOX1/CD28 network was constructed (Fig. 2A).

### Immune cell patterns in CAVD

**Fig. 3:**
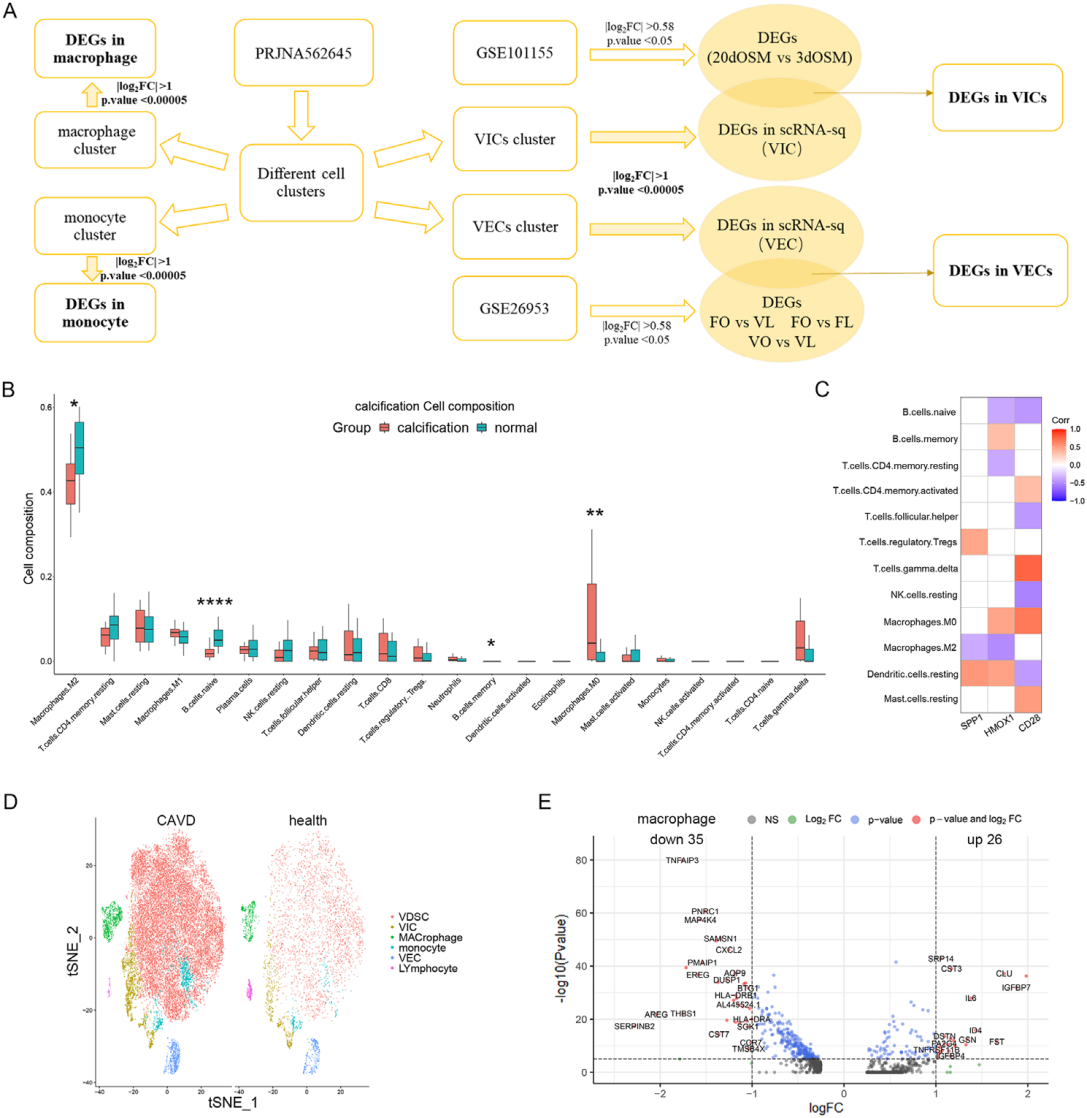
Immune Cell Patterns in CAVD. (**A**) Workflow of the scRNA-seq data analysis. (**B**) Pattern of immune cell infiltration in CAVD. (**C**) Correlation analysis between expression levels of SPP1, HMOX1, CD28 and immune cells. (**D**) The t-SNE projection cluster scatter diagram of the combined CAVD and health samples in PRJNA562645, with 6 identified cell types. (**E**) Volcano plot of DEGs in macrophages between CAVD and health. *p < 0.05; **p < 0.01; ****p < 0.0001

Immune cells play important roles in CAVD [21]. In the present work, the characteristics of some immune cells involved in CAVD were evaluated on the immune infiltration and single cell level (Figs. 1 and 3 A). The immune infiltration analysis showed that the levels of M0 macrophages and memory B cells were significantly increased, while levels of M2 macrophages and naive B cells were significantly decreased in CAVD compared with normal controls (Fig. 3B). The correlation analysis results show that M0 macrophages were positively correlated with γδ T cells (r = 0.52, p < 0.05), while being negatively correlated with naive B cells (r = − 0.44, p < 0.05), M2 macrophages (r = − 0.54, p < 0.05) and resting NK cells (r = − 0.38, p < 0.05) (Figure S4 A). The correlation between SPP1, HMOX1, CD28 and immune cell infiltration in CAVD was investigated by Spearman correlation analysis. The results show that the expression level of these three up-regulated genes was positively correlated with increased levels of immune cells (M0 macrophages and memory B cells) (Fig. 3C).

Single-cell RNA sequencing is a fast-evolving and powerful technology to reveal the potential mechanisms and pivotal cells involved in special biological processes and diseases. To further understand the roles that immune cells play in CAVD, we mine and reanalyze scRNA-seq data from publicly available resources uploaded by Xu et al. [22]. The cells in the scRNA-seq were divided into six cell clusters, including macrophages, monocytes, lymphocytes, VICs, VECs and valve-derived stromal cells (Fig. 3D). Due to the low number of lymphocytes, we selected macrophage and monocyte clusters for differential analysis. The results show that there were 61 DEGs in macrophages and 167 DEGs in monocytes between CAVD and healthy controls (Fig. 3E, Figure S4B and Table S10-S11).

### Identification of DEGs in VICs and VECs

**Fig. 4:**
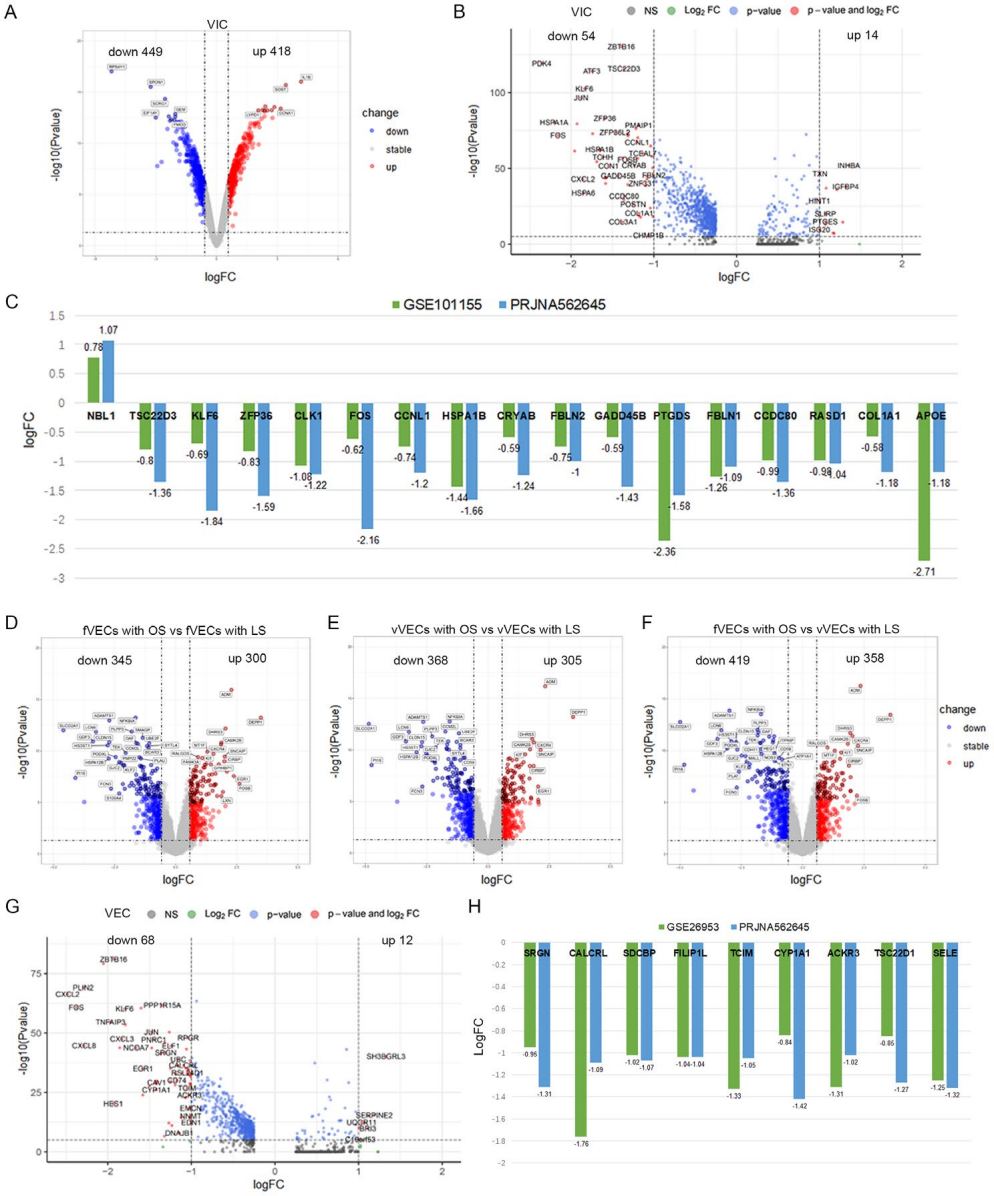
CAVD-related DEGs in VICs and VECs. (**A**) Volcano plot of the DEGs between VICs cultured with OSM for 20 and 3 days (from GSE101155). (**B**) Volcano plot of DEGs in VICs between CAVD and health (from PRJNA562645). (**C**) The intensity of the differential expression of the overlapped DEGs in VICs. (**D-F**) Volcano plot of the DEGs in fVECs and vVECs between OS and LS (from GSE26953). (**G**) Volcano plot of DEGs in VECs between CAVD and health (from PRJNA562645). (**H**) The intensity of the differential expression of the overlapped DEGs in VECs.

VICs and VECs are important cells in the aortic valve and are closely related to the occurrence and development of CAVD. We identified the possible CAVD-related DEGs in VICs in datasets GSE101155 and PRJNA562645 (Fig. 3A). In GSE101155, the researchers induced the differentiation of VICs to an osteogenic phenotype by the application of osteogenic medium (OSM). We found that the gene expression profile of VICs cultured with OSM for 3 days was similar to that of VICs cultured with Dulbecco’s modified Eagle Medium (DMEM), indicating that the osteogenic induction for 3 days was not enough to induce the transformation of the VICs’ phenotype (Figure S5 A). Therefore, we identified 418 up-regulated and 449 down-regulated genes by comparing 20-day with 3-day cultured VICs in OSM (Fig. 4A and Table S12). For the scRNA-seq PRJNA562645, 68 DEGs were identified from the VIC cluster (Fig. 4B and Table S13). Finally, a total of 17 DEGs were obtained by overlapping the microarray and scRNA-seq data and excluding the inconsistently expressed genes (Figure S5 D). TSC22D3, KLF6, ZFP36, CLK1, FOS, CCNL1, HSPA1B, CRYAB, FBLN2, GADD45B, PTGDS, FBLN1, CCDC80, RASD1, COL1A1 and APOE were down-regulated, while NBL1 was up-regulated (Fig. 4C).

Hemodynamic abnormality was considered to be involved in the development of CAVD. GSE26953 collected gene expression profiles of VECs from the fibrosa (fVECs) and ventricularis (vVECs) under laminar (LS) and oscillatory (OS) shear stress. Firstly, we compared the gene expression profiles of the two kinds of VECs under the same shear stress, and found that the gene expression profiles overlapped mostly either with LS or OS (Figure S5 B-C). Secondly, we analyzed the gene expression profiles of the same cells under different shear stress. The results show that compared with LS, 300 genes were up-regulated and 345 genes down-regulated in fVECs, and 305 genes were up-regulated and 368 genes down-regulated in vVECs under OS (Fig. 4D-E and Table S14-15). Meanwhile, considering the VECs under physiological conditions in vivo, we compared the gene expression of fVECs with OS and vVECs with LS, and a total of 777 DEGs were identified, among which 358 were up-regulated and 419 down-regulated (Fig. 4F and Table S16). Next, we also identified the DEGs in the VEC cluster in PRJNA562645 (Fig. 4G and Table S17). Finally, after excluding the inconsistently expressed genes, nine genes (SRGN, CALCRL, SDCBP, FILIP1L, TCIM, CYP1A1, ACKR3, TSC22D1 and SELE) were obtained by overlapping the microarray and scRNA-seq results. Additionally, all of these genes were down-regulated (Figure S5 E and Fig. 4H).

### Identification of CAVD-related DEGs between BAVs and TAVs

**Fig. 5:**
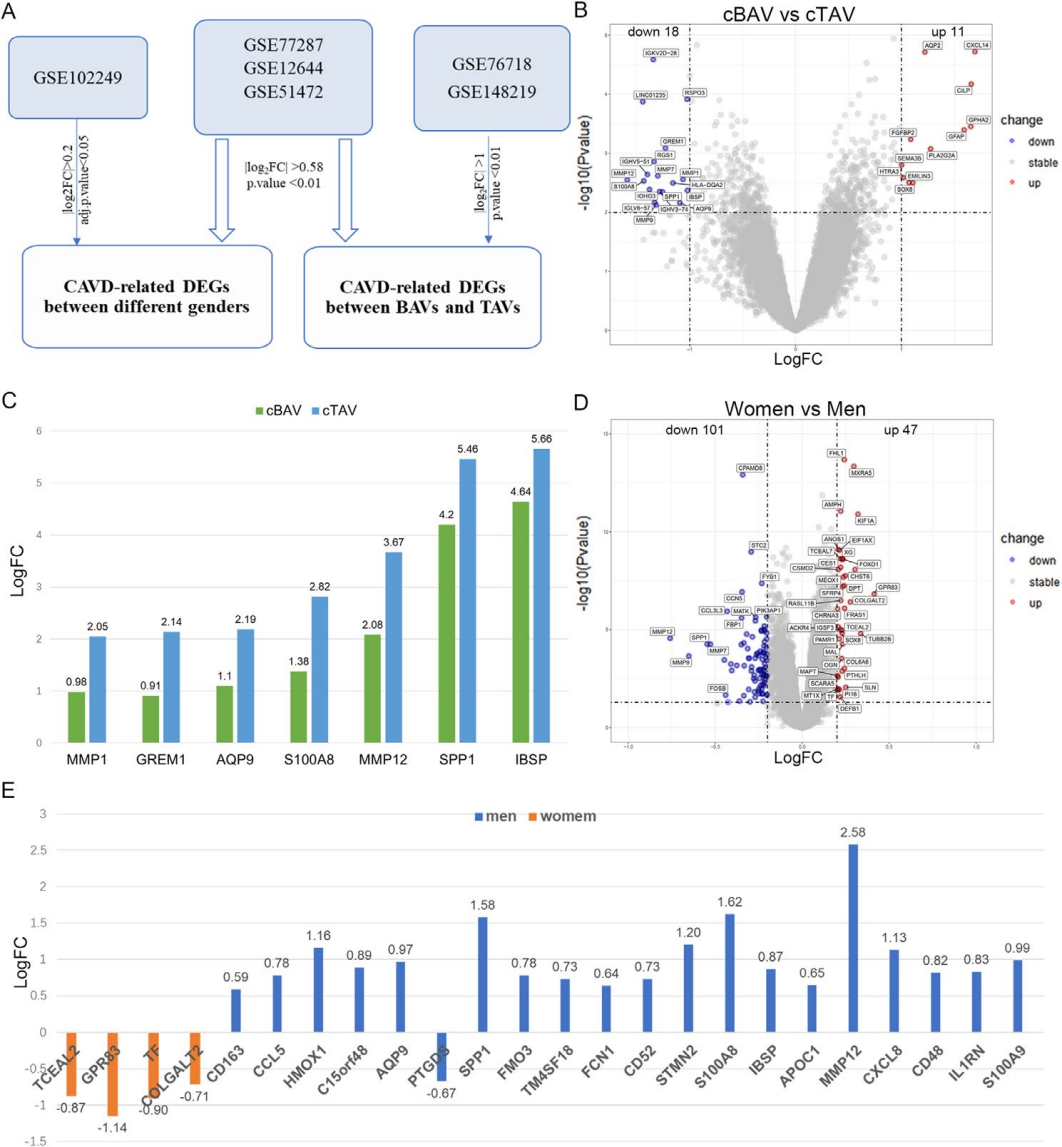
CAVD-related DEGs between BAVs and TAVs, and different genders. (**A**) Workflow of the differential analysis. (**B**) Volcano plot of the DEGs between calcified cBAVs and cTAVs from GSE76718 and GSE148219. (**C**) The intensity of the differential expression of the overlapped DEGs between cBAVs and cTAVs. (**D**) Volcano plot of the DEGs between women and men with CAVD (from GSE102249). (**E**) The intensity of the differential expression of gender-specific DEGs in CAVD.

BAV is a common aortic valve malformation, and is more prone to calcification due to different shear stresses [18], but the specific pathogenic mechanisms are unclear. In this work, we identified CAVD-related DEGs between cBAVs and cTAVs from datasets GSE76718 and GSE148219 (Fig. 5A). In total, 29 DEGs were identified (Fig. 5B and Table S18), and 7 DEGs related to CAVD between BAVs and TAVs were obtained by overlapping them with the 167 DEmRNAs identified from the three microarray datasets above (GSE77287, GSE12644 and GSE51472) (Figure S5 F). AQP9, S100A8, MMP12, SPP1 and IBSP were all up-regulated in CAVD, and had bigger enhancements in cTAVs when compared to cBAVs, but MMP1 and GREM1 were up-regulated only in cTAVs (Fig. 5C).

### Identification of CAVD-related DEGs between different genders

Previous evidences indicate that aortic valve calcification follows different mineralization pathways between different genders [20]. As shown in Fig. 5A, we identified CAVD-related DEGs between different genders by overlapping the gender-specific DEGs with CAVD-related DEGs (GSE77287, GSE12644 and GSE51472). With an adjusted p. value < 0.05 and |log_2_FC| > 0.2 as the cut-off criteria, 47 up-regulated DEGs in women and 101 up-regulated DEGs in men were identified from GSE102249 (Fig. 5D and Table S19). Then, we overlapped these DEGs with the 167 DEmRNAs identified from the three microarray datasets above. Finally, 24 CAVD-related DEGs between different genders were obtained (Figure S5 G). Among them, TCEAL2, GPR83, TF, COLGALT2 and PTGDS were down-regulated in CAVD, and levels were decreased more in men, except for PTGDS. CD163, CCL5, HMOX1, C15orf48, AQP9, SPP1, FMO3, TM4SF18, FCN1, CD52, STMN2, S100A8, IBSP, APOC1, MMP12, CXCL8, CD48, IL1RN and S100A9 were up-regulated in CAVD and levels were increased more in men (Fig. 5E).

## Discussion

Recent studies found that CAVD is a progressive disease, from fibrosis and sclerosis to chronic inflammation and then to lobular calcification, which eventually leads to aortic valve stenosis [23]. However, its molecular mechanisms are still not fully understood. With the discovery of the differential expression of ceRNAs in CAVD, the establishment of the circRNA/lncRNA–miRNA–mRNA regulatory network may help to clarify the pathogenesis of CAVD andfind therapeutic targets.

Using the bioinformatics methods, a hsa-circ-0073813/hsa-circ-0027587–hsa-miR-525-5p–SPP1/HMOX1/CD28 network was constructed and verified by qRT-PCR. The 3 hub genes were verified by independent data sets at the same time. The first hub gene is secreting phosphoprotein 1 (SPP1), also known as bone salivary protein 1, and early T lymphocyte activation 1 (ETA-1), controlling cell growth, proliferation, apoptosis, and migration [24]. SPP1 was up-regulated in CAVD compared to normal controls, and this result is consistent with previous studies [25, 26]. SPP1 promotes the growth of fibroblasts and mesenchymal stem cells and type I collagen production, which is involved in the fibrosis of bone marrow and heart [27, 28]. In addition, macrophages can secrete SPP1 through exosomes, which promotes the transformation of fibroblasts into myofibroblasts and promotes pulmonary fibrosis [29]. Additionally, it has been confirmed that SPP1 can promote the osteogenic differentiation of VICs [28]. In addition, SPP1 contributes to immune cell migration, cytokine release, and calcium deposition [30]. All of this evidence suggests the crucial role of hub gene SPP1 in CAVD.

The second hub gene, heme oxygenase 1 (HMOX1 or HO-1), is an important enzyme in heme oxygenase metabolism. HMOX1 can enhance osteogenic differentiation by activating PI3K/Akt, WNT/β-catenin, and P38 or inhibiting ERK1/2 signaling pathways [31]. The expression of HMOX1 is reduced in humans and mice with osteoarthritis, aggravating cartilage damage and bone remodeling [32]. In addition, HMOX1-dependent myocardial inflammation and fibrosis were closely associated with doxorubicin-induced cardiotoxicity [33]. However, other studies have found that HMOX1 can inhibit the calcification of human VICs in vitro, and the up-regulation of HMOX1 can relieve oxidative stress and vascular calcification in chronic kidney disease [34, 35]. Our study found that HMOX1 expression was up-regulated in calcified aortic valves, but interestingly was down-regulated in VECs and VICs cultured in vitro. The aortic valve contains a variety of cells, and the expression level of HMOX1 in different cells may be different. Furthermore, our results suggest that HMOX1 may play a role in CAVD independent from VECs and VICs.

The last hub gene is CD28, which is a co-stimulatory molecule expressed on the surface of T lymphocytes, and that plays an important role in T cell activation. The pathology of CAVD is similar to atherosclerosis; for example, T cell infiltration under the endothelial cells. Activated T lymphocytes release cytokines, which promote extracellular remodeling by increasing the production of metalloproteinases [36, 37]. On the other hand, T lymphocytes and their products are considered to be key regulators in the formation, longevity and activation of osteoclasts and osteoblasts [38]. In addition, MacGrogan et al. found that CD28 was specific to aortic valve calcification and may act as a circulating biomarker for CAVD [39]. In short, the hub gene CD28 was up-regulated in CAVD and may be involved in the pathogenesis of CAVD.

There have been many studies focused on the roles of ncRNAs in CAVD. In this work, hsa-circ-0073813 and hsa-circ-0027587 were finally included in the ceRNA network. Unfortunately, these two circRNAs have not been reported. They may affect CAVD by inhibiting their target miRNAs and thereby affecting the three hub genes identified by us, as circRNAs exert their gene regulation function through miRNA sponging [40], but their specific mechanisms need to be further studied. A total of 45 DEmiRNAs were identified in our analysis, but only has-miR-525-5p was finally verified in the clinical CAVD samples. It was down-regulated during chondrogenic differentiation and regulates HOXD10 [41], but how is has-miR-525-5p involved in CAVD needs more study. In addition, we identified 397 annotated DElncRNAs, and found that two of them, LINC01018 and MAPT-IT1, were included in the lncRNA–miRNA–hub gene network. LINC01018 is a metabolism-related lncRNA that regulates the expression of genes in fatty acid metabolism, such as ADH1C, CYP4A11, CYP4A22, BDH1 and ALDH5A1 [42]. MAPT-IT1 was up-regulated in breast cancer [43], and up-regulated in CAVD in this work, but its mechanism is still unknown. In short, the circRNA/lncRNA–miRNA–hub gene network constructed in this work may improve research into the mechanisms of CAVD and provide potential drug targets.

Functional enrichment analysis of DEmRNAs in the ceRNA network helps us to understand the potential biological functions. According to our results, these DEmRNAs were mainly involved in the “chemokine signaling pathway”, “cytokine-cytokine receptor interaction”, “Toll-like receptor signaling pathway” and “WNT signaling pathway”. As is well known, the pathological mechanisms of CAVD involve inflammation, fibrosis and mineral deposition. The triggering stage of aortic valve calcification resembles atherosclerosis, with lipid infiltration and inflammatory responses due to endothelial damage. Inflammation and collagen deposition promote progressive fibrosis and calcification [14]. The inflammatory response also promotes the transformation of VICs to an osteoblastic phenotype [44]. Our results suggest the potentially important pathways in inflammation and fibro-calcification during the pathogenesis of CAVD.

We also analyzed the immune cells in CAVD. Immune infiltration analysis showed results consistent with those of previous research, suggesting that levels of M0 macrophages and memory B cells were increased, while levels of M2 macrophages and naive B cells decreased in CAVD [45, 46]. In addition, our results show that the hub genes in the ceRNA network were positively correlated with the increased level of immune cells. The aortic valve contains immune cells such as macrophages, T lymphocytes and B lymphocytes, which were increased in calcified specimens [47]. One study reported that VICs treated with conditioned medium of M1 macrophages showed a tendency toward osteogenic differentiation [48]. Additionally, macrophages can secrete SPP1, which is involved in CAVD [49]. Moreover, the enrichment of B lymphocytes in valves is associated with increased severity of disease [50]. In this work, we identified three immune cell clusters from scRNA-seq data, including macrophages, lymphocytes and monocytes, and further identified DEGs in macrophage and monocyte clusters. Through literature reviewing, we found that ceRNAs may also participate in CAVD through immune cells. Studies have found that the expression level of has-miR-296-a was positively correlated with the serum levels of IL-6 and TNF-α, which were increased in patients with coronary heart disease [51]. In our study, the gene expression level of IL-6 in macrophage was also elevated in CAVD group. In addition, P-selectin and junctional adhesion molecule B inhibit monocyte adhesion and participate in leukocyte infiltration in human umbilical vein endothelial cells, whereas has-miR-27a inhibits this process [52]. In conclusion, the discovery of these DEGs in immune cells provides insight into the role of immunity and inflammation in CAVD, as well as future potential drug targets.

VICs and VECs are the most important cells in the aortic valve, and their phenotypic and functional changes have a significant impact on CAVD. In this work, we identified genes involved in calcification that were differentially expressed in both microarray and single-cell sequencing. APOE is involved in lipoprotein metabolism and participates in the activation of lipolytic enzymes, which is closely related to the occurrence and development of atherosclerosis. In addition, APOE was also involved in immune regulation and the regeneration of nerve tissue [53, 54]. ZFP36 and PTGDS have anti-inflammatory properties, which reduce vascular inflammation and prevent atherosclerosis [55, 56]. TSC22D3 mediated glucocorticoid-induced osteogenic differentiation but inhibited the differentiation of mesenchymal stem cells into adipocytes [57]. The osteogenic differentiation of VICs is an important process of CAVD, and TSC22D3 may also mediate this process. A previous study indicated that FBLN2 may be a candidate effector gene for Notch signaling to promote the development of the endocardium (including cardiac valves) [58]. These DEGs have a variety of biological functions, and they participate in CAVD through inducing osteogenic differentiation, fibrosis or other mechanisms, which still need to be further studied.

Like other endothelial cells, VECs form a protective layer for the aortic valve and are directly vulnerable to abnormal shear stress, which may be the initiating mechanism of aortic valve calcification. Studies have shown that high glucose can induce biological processes such as vascular endothelial cell proliferation and migration, thereby accelerating plaque formation and vascular stenosis [59, 60]. However, some miRNAs, including miR-181b, miR-204, miR-520a and miR-20a, can prevent this process by down-regulating the expression of HCK, β-ARR2, ERK, FAK and VASP genes [61–63]. These interacting miRNA-mRNA pairs may also be involved in CAVD by regulating the cellular function of VECs. However, the changes and mechanisms of VECs during CAVD remain unclear. In our study, SRGN, CALCRL, SDCBP, FILIP1L, TCIM, CYP1A1, ACKR3, TSC22D1 and SELE were identified as possible effector genes in the calcification of VECs. SRGN and CYP1A1, may be key genes in the endothelial cell response to shear stress in atherosclerosis [64, 65]. Additionally, in atherosclerosis, activated G_s_-coupled receptor calcitonin receptor-like receptor (CALCRL) induces anti-inflammatory signals and reduces endothelial inflammation [66]. However, ACKR3 and SELE drive atherosclerosis by promoting immune cell adhesion to the vascular endothelium [67, 68]. Filamin A interacting protein 1-like (FILIP1L) is an inhibitor of the WNT pathway. It inhibits WNT/β-catenin signaling and regulates the epithelial–mesenchymal transition and extracellular matrix synthesis [69]. In conclusion, these genes are mainly expressed in endothelial cells and in the response to shear stress, cell damage and the mediated inflammatory response, and these mechanisms may also be involved in the progression of CAVD.

It has been reported that more than 50% patients with severe aortic stenosis requiring AVR have BAVs [70]. It is of great significance to interpret DEGs related to CAVD between BAVs and TAVs. The imbalanced expression of matrix metalloproteases (MMPs) can accelerate leaflet reconstruction and valvular degeneration by changing the structural composition of the valve matrix, thus leading to valvular dysfunction [71]. In our study, MMP1 was mainly involved in cBAVs, while MMP12 was involved in cTAVs. It has been found that shear stress can induce the up-regulation of MMP1, thus promoting the occurrence and progression of atherosclerosis [72]. MMP12 degrades extracellular matrix components, participates in tissue remodeling processes, and is involved in acute and chronic inflammatory diseases, such as pulmonary fibrosis [73]. In addition, in our study, the expression of SPP1 was up-regulated in CAVD tissues, but down-regulated in osteogenesis induced VICs, and stable in VECs with shear stress, indicating that the expression of SPP1 varied in different cells and with different interventions, and the mechanism of its participation in CAVD was complex. Furthermore, secreted bone Morphogenetic protein antagonist, Gremlin1 (GREM1), can inhibit the osteogenic transformation of adipocytes, and some scholars believe that the expression of GREM1 can be a marker of activated myofibroblasts in cancer matrix or scar tissue [74]. Moreover, IBSP can inhibit osteoblast differentiation and promote the osteolytic metastasis of breast cancer [75]. These studies suggest that GREM1 and IBSP may be involved in the calcification of the aortic valve. The roles of calcification-related DEGs identified in our work are complex, and their specific mechanisms need to be further studied.

The gender differences in CAVD have been widely recognized, but the underlying molecular mechanism remains unclear. A previous work identified DEGs among patients with CAVD of different genders [76]. In our analysis, we intersected these gender-differentiated genes with CAVD-related DEGs, and finally obtained genes associated with CAVD between different genders. This work allows us to understand gender differences in CAVD at the molecular level. Interestingly, the two hub genes, SPP1 and HMOX1, were also found here. In fact, gender differences in SPP1 expression were found in both human and animal disease models, with higher expression levels in males than in females, which was consistent with our results. In addition, SPP1 allele polymorphism was found to also be associated with gender [77, 78]. For HMOX1, an in vitro study showed that the down-regulation of estrogen-associated receptor α could up-regulate HMOX1 and inhibit the calcification of VICs [34]. These results suggest that gender differences are involved in the occurrence and development of CAVD. Further studies are needed to interpret the roles of other DEGs found here for CVAD.

## Conclusions

It is of great significance to understand the pathogenesis of CAVD for its prevention and treatment. We identified the differentially expressed circRNAs, lncRNAs, miRNAs and mRNAs in CAVD and established a circRNA/lncRNA–miRNA–mRNA network. After independent data sets and qRT-PCR verification, a hsa-circ-0073813/hsa-circ-0027587–hsa-miR-525-5p–SPP1/HMOX1/CD28 network was finally constructed, which contribute to a deeper understanding of CAVD and provide possible targets for the treatment of CAVD in the future. At the same time, we also conducted subgroup analysis for DEGs among different cell types in CAVD, such as immune cells, VICs and VECs, in addition to different aortic valve morphologies and genders. The discovery of these DEGs will help us understand the differences between these characteristics in CAVD at the molecular level and provide evidence for future personalized therapy.

## Materials and methods

### RNA expression data Collection and Information

The circRNA and lncRNA expression profiles were collected from GSE155119, including 3 CAVD cases and 3 normal controls. The miRNA expression profile was obtained from GSE87885, containing 2 CAVD cases and 3 normal controls. The mRNA data were obtained from 3 microchips, GSE77287, GSE12644 [79] and GSE51472 [80], including 18 CAVD cases and 18 normal controls in total. GSE101155 [81] and GSE26953 [15] were used to identify the DEGs in VICs and VECs, respectively. In GSE102249 [82], 120 male and 120 female CAVD patients were used to search for sex-specific genes. GSE76718 [83], containing 10 cBAVs and 9 cTAVs, and GSE148219 [39], containing 5 cBAVs and 7 cTAVs, were used to identify the DEGs between cBAVs and cTAVs. These datasets were downloaded from the GEO database (https://www.ncbi.nlm.nih.gov/geo/) [84]. The scRNA-seq data was obtained from NCBI (https://www.ncbi.nlm.nih.gov/bioproject/) [85], and the persistent ID was PRJNA562645, containing 4 CAVD cases and 2 healthy controls [22]. The basic details of these RNA expression data are shown in Table 1.

**Table 1:** Basic information of different datasets

Profile	Platform	RNA type	Experiment type	Sample
GSE155119	GPL26192	circRNA lncRNA mRNA	Expression profiling by array	3 CAVD / 3 normal
GSE87885	GPL22555	miRNA	Non-coding RNA profiling by array	2 CAVD / 3 normal
GSE77287	GPL16686	mRNA	Expression profiling by array	3 CAVD / 3 normal
GSE12644	GPL570	mRNA	Expression profiling by array	10 CAVD / 10 normal
GSE51472	GPL570	mRNA	Expression profiling by array	5 CAVD / 5 normal
GSE101155	GPL10904	mRNA	Expression profiling by array	VICs
GSE26953	GPL6947	mRNA	Expression profiling by array	VECs
GSE102249	GPL10558	mRNA	Expression profiling by array	120 female /120 male CAVD
GSE76718	GPL11154	mRNA	Expression profiling by high throughput sequencing	10 cBAVs / 9 cTAVs / 8 normal
GSE148219	GPL10999	mRNA	Expression profiling by high throughput sequencing	5 cBAVs / 7 cTAVs / 8 normal
PRJNA562645	Illumina HiSeq Xten	mRNA	Single-cell RNA sequencing	4 CAVD / 2 normal

CAVD, calcific aortic valve disease; cBAVs, calcified bicuspid aortic valves; cTAVs, calcified tricuspid aortic valves; VICs, valve interstitial cells; VECs, valvular endothelial cells.

### Identification of DEcircRNAs, DElncRNAs, DEmiRNAs, and DEmRNAs in CAVD and CAVD-related cells

The RNA expression matrix and platform files of circRNA, lncRNA, miRNA and mRNA were downloaded using the “GEO query” package of R software (Version 4.1.2) [86]. For multiple datasets, batch normalization was performed by the “sva” package after merging the microarray data. Then, PCA was used to detected and excluded the abnormal samples to ensure the data could be used for subsequent analysis. Finally, differential analysis was performed by “limma” package of R software. Different p values and |log_2_FC| were set as the thresholds (Fig. 1A), and the DEcircRNAs, DElncRNAs, DEmiRNAs and DEmRNAs were collected. The results were visualized as heat maps and volcanic maps using the “ggplot2” package.

For the scRNA-seq data, after quality control and standardization of the original data, PCA was performed. Then, the “ElbowPlot” was used to plot the distribution points map; “Shared Nearest neighbor” and “Find Clusters” were used to perform unbiased clustering of cells; t-distributed stochastic neighbor embedding (t -SNE) was used to visualize dimensionality reduction. Next, “FindAllMarkers” was used to identify DEGs between different cell types, and the cells were divided into several clusters based on the differential and characteristic genes. Finally, “Seurat” package was used for differential analysis. Threshold = 0.25 and test.use = “Wilcox” were set as the thresholds. The DEGs in different cell clusters between CAVD cases and healthy controls were collected [22].

### Construction of the circRNA/lncRNA–miRNA–mRNA network in CAVD

Firstly, circPrimer (Version 2.0) was used to select the exon DEcircRNAs for further analysis [87]. CircBank (http://www.circbank.cn/) was used to predict the interactions between miRNAs and the exon DEcircRNAs [88]. Then, the predicted miRNAs and DEmiRNAs obtained from GSE87885 were intersected to obtain the shared miRNAs. Next, the DEmiRNAs in the intersection were typed into TargetScan Human (Version 7.2) (http://targetscan.org/) and miRDB (http://www.mirdb.org/), respectively, to identify their interaction mRNAs [89, 90]; and the mRNAs that could be predicted in both databases were intersected with the DEmRNAs obtained from GSE77287, GSE12644 and GSE51472. Finally, we integrated the DEcircRNAs–DEmiRNAs and DEmiRNAs–DEmRNAs interaction pairs and constructed a circRNA–miRNA–mRNA network, which was visualized by the “ggalluvial” package of R software.

DEmiRNAs identified from GSE87885 were studied in StarBase (https://starbase.sysu.edu.cn/) to search for interaction lncRNAs [91], as well as for mRNAs in TargetScan and miRBD. Only the interaction mRNAs predicted in both TargetScan and miRBD were collected. Then, the interaction lncRNAs and mRNAs were intersected with the DElncRNAs and DEmRNAs identified from microarray data. Finally, lncRNAs, miRNAs and mRNAs in the intersections were integrated to construct the lncRNA–miRNA–mRNA interaction network, which was visualized with the “ggalluvial” package.

### Functional Enrichment and Pathway Analysis

Gene ontology (GO) describing genes in terms of CC, MF and BP, is the largest standardized gene function classification system [92]. Kyoto Encyclopedia of Genes and Genomes (KEGG) is a visual systematic analysis database for analyzing the biological signaling pathways of genes [93]. We used the “cluster Profiler” package for GO annotation and KEGG pathway enrichment analysis. p. value < 0.05 and more than 3 DEGs enrichment were considered statistically significant. The results were visualized by “ggplot2” package.

### PPI Network Construction and Hub Gene Identification

The online database, Search Tool for the Retrieval of Interacting Genes (STRING) (version11.5) (https://www.string-db.org/), helps us find the core hub genes from a large number of regulatory genes by studying the relationship between proteins [94]. Set the confidence > 0.4, and the PPI network were visualized using Cytoscape (version 3.8.2) [95]. The MCC algorithm of the cytoHubba with a higher accuracy in identifying key nodes in the PPI network was used to extract hub genes [96]. Next, hub genes and the interaction circRNAs, lncRNAs and miRNAs were selected from the ceRNA network to construct a circRNA/lncRNA–miRNA–hub gene network according to the ceRNA hypothesis.

### Verification of hub genes, miRNAs and circRNAs

GSE148219 and GSE76718, which analyzing the expression profiles of 15 cBAVs, 16 cTAVs and 18 normal controls in total by high-throughput sequencing, were used to validate the hub genes. The basic details of the data and the thresholds are described above (Fig. 5A; Table 1). After merging these two datasets, we identified the DEGs of cBAV and cTAV compared with the normal controls. Then, we verified the hub genes. Additionally, the hub genes, miRNAs and circRNAs were further verified by qRT-PCR.

### Human Specimen Collection and Ethics

Aortic valve specimens were collected from three patients with severe aortic valve calcification and three patients with aortic valve regurgitation but no significant calcification in AVR surgery and preserved at − 80◦C for qRT-PCR. The clinical features of the individuals are shown in Table S20. Informed consents were signed by all subjects before the study. The study was conducted according to the Declaration of Helsinki and approved by the Ethics Committee of Tongji Medical College, Huazhong University of Science and Technology (IEC Approval Number: UHCT-IEC-SOP-016-03-01).

### qRT-PCR

Firstly, Trizol reagent (Takara, Beijing, China) was used to isolate the total RNA from aortic valve tissues according to the manufacturer’s protocol. Then, reversely transcription was performed with HiScript® III RT SuperMix for qPCR (+ gDNA wiper) (Vazyme, Nanjing, China) or miRNA 1st Strand cDNA Synthesis Kit (by stem-loop) (Vazyme, Nanjing, China). The qRT-PCR was performed with ChamQ SYBR qPCR Master Mix (Vazyme, Najing, China) in the Real-time PCR Detection System (Bio-Rad, United States). GAPDH or U6 were used as endogenous controls and the 2^−ΔΔCT^ method was used to analysis the RNAs relative expression level. The primers purchased from Tsing Biotechnology Co., Ltd (China) and the sequence information is shown in Table S21.

### Immune Cell Infiltration of CAVD

The CIBERSORTx database (https://cibersortx.stanford.edu/) estimating the abundance of different cells by gene expression data, was used to estimate the immune cell infiltration in CAVD [97]. Then, the correlation between different immune cells, hub genes and immune cells related to CAVD were analyzed by Spearman correlation analysis. The “ggplot” and “ggcorrplot” packages were used to visualize the results.

## Electronic supplementary material

Below is the link to the electronic supplementary material.

**Supplementary Material 1: Fig S1**. Identification of DEcircRNAs, DElncRNAs, DEmiRNAs and DEmRNAs

**Supplementary Material 2: Fig S2**. Sankey diagram of the ceRNA network in CAVD

**Supplementary Material 3: Fig S3**. Functional enrichment, pathway analysis and the PPI network construction

**Supplementary Material 4: Fig S4**. Immune cell patterns in CAVD

**Supplementary Material 5: Fig S5**. Identification of CAVD-related DEGs between BAVs and TAVs, and different genders

**Supplementary Material 6: Table S10**. DEGs identified from the macrophage cluster in PRJNA562645

**Supplementary Material 7: Table S11**. DEGs identified from the monocyte cluster in PRJNA562645

**Supplementary Material 8: Table S12**. DEGs in VICs identified from GSE101155

**Supplementary Material 9: Table S13**. DEGs identified from the VIC cluster in PRJNA562645

**Supplementary Material 10: Table S14**. DEGs between fVECs with OS and fVECs with LS (from GSE26953)

**Supplementary Material 11: Table S15**. DEGs between vVECs with OS and vVECs with LS (from GSE26953)

**Supplementary Material 12: Table S16**. DEGs between fVECs with OS and vVECs with LS (from GES26953)

**Supplementary Material 13: Table S17**. DEGs identified from the VEC cluster in PRJNA562645

**Supplementary Material 14: Table S18**. DEGs between cBAVs and cTAVs from GSE76718 and GSE148219

**Supplementary Material 15: Table S19**. DEGs between different genders in GSE102249

**Supplementary Material 16: Table S1**. DEcircRNAs identified from GSE155119 and their types

**Supplementary Material 17: Table S20**. Clinical characteristics of the patients collected for qRT-PCR

**Supplementary Material 18: Table S21**. Primer sequences information

**Supplementary Material 19: Table S2**. DElncRNAs identified from GSE155119

**Supplementary Material 20: Table S3**. DEmiRNAs identified from GSE87885

**Supplementary Material 21: Table S4**. DEmRNAs identified from merged GSE77287,GSE12644 and GSE51472

**Supplementary Material 22: Table S5**. MiRNAs in the intersection of DEmiRNAs and predicted miRNAs by DEcircRNAs

**Supplementary Material 23: Table S6**. MRNAs in the intersection of DEmRNAs and predicted mRNAs by DEmiRNAs

**Supplementary Material 24: Table S7**. LncRNAs and mRNAs in the intersection of DElncRNAs and DEmRNAs with the DEmiRNAs prediction results

**Supplementary Material 25: Table S8**. The circRNA/lncRNA-miRNA-mRNA network in CAVD

**Supplementary Material 26: Table S9**. The entries of GO and KEGG enrichment analysis

## Data Availability

The datasets generated and/or analyzed during the current study are available in the GEO (https://www.ncbi.nlm.nih.gov/geo/) and NCBI repository (https://www.ncbi.nlm.nih.gov/bioproject/). The scripts and codes used in this work can be downloaded in GiHub at https://github.com/1291988308/RNA.git.

## Abbreviations

CAVD: Calcific aortic valve disease
VIC: Valve interstitial cell
VEC: Valvular endothelial cell
ceRNA: Competitive endogenous RNA
DEG: Differentially expressed gene
AVR: Aortic valve replacement
ncRNA: Non-coding RNA
miRNA: Micro RNA
lncRNA: Long non-coding RNA
circRNA: Circular RNA
scRNA-seq: Single-cell RNA sequencing
BAV: Bicuspid aortic valve
TAV: Tricuspid aortic valve
PCA: Principal component analysis
t -SNE: t-distributed stochastic neighbor embedding
GO: Gene ontology
BP: Biological process
CC: Cellular component
MF: Molecular function
KEGG: Kyoto Encyclopedia of Genes and Genomes
PPI: Protein-Protein Interaction
STRING: Search Tool for the Retrieval of Interacting Genes
OSM: Osteogenic medium
DMEM: Dulbecco’s modified Eagle Medium
LS: Laminar shear stress
OS: Oscillatory shear stress
qRT-PCR: Quantitative real-time polymerase chain reaction
