# Supplementary material for: CircRNA/lncRNA–miRNA–mRNA network and gene landscape in calcific aortic valve disease
Source: BMC Genomics. 2023 Jul 25;24:419. doi: 10.1186/s12864-023-09441-y (PMC10367311; doi:10.1186/s12864-023-09441-y)
Supplement: Supplementary file 1 — Supplementary Material 1: Fig S1. Identification of DEcircRNAs, DElncRNAs, DEmiRNAs and DEmRNAs [file 12864_2023_9441_MOESM1_ESM.pdf]

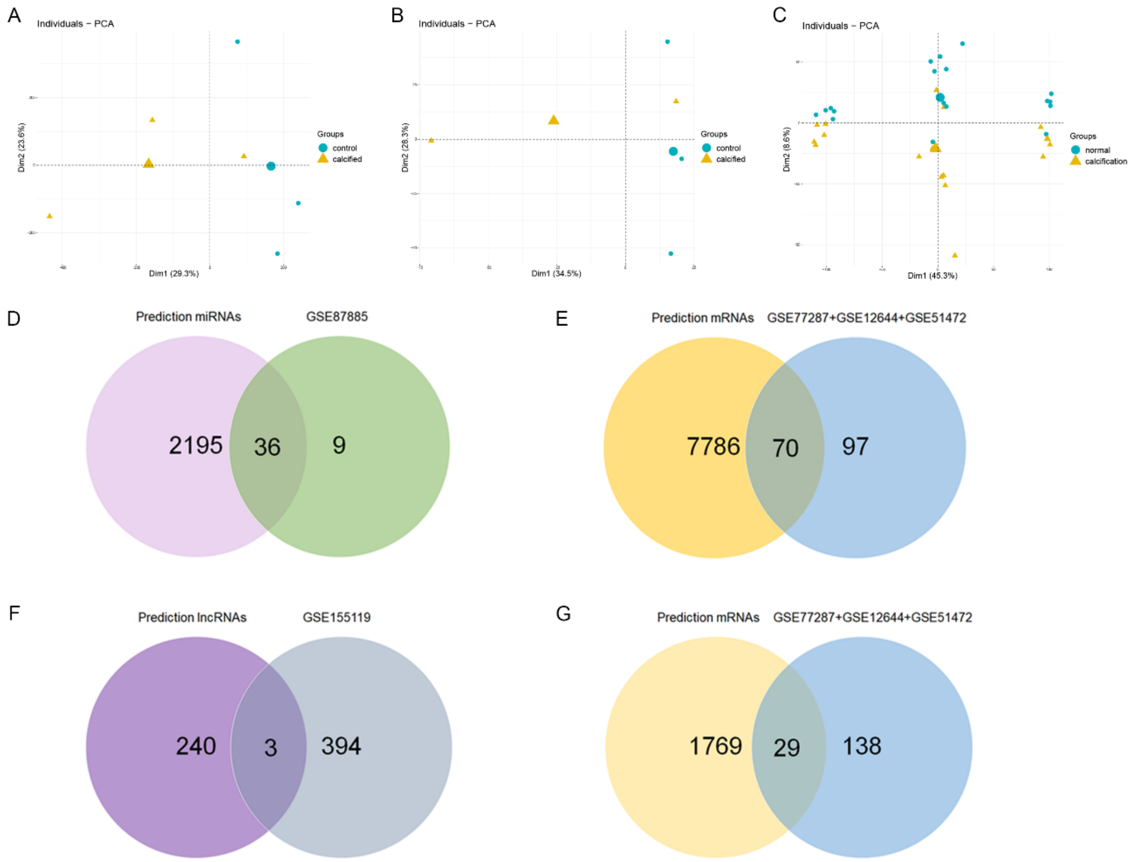

FIGURE S1 | Identification of DEcircRNAs, DElncRNAs, DEmiRNAs and DEmRNAs. (A-C) The PCA of GSE155119 (A) GSE87885 (B) and merged GSE77287, GSE12644 and GSE51472 (C). (D) Venn diagram of DEmiRNAs intersected with miRNA predicted by DEcircRNAs. (E) Venn diagram of DEmRNAs intersected with mRNA predicted by DEmiRNAs. (F) Venn diagram of DElncRNAs intersected with lncRNAs predicted by DEmiRNAs. (G) Venn diagram of DEmRNAs intersected with mRNA predicted by lncRNA-interacted DEmiRNAs.
