# Supplementary material for: CircRNA/lncRNA–miRNA–mRNA network and gene landscape in calcific aortic valve disease
Source: BMC Genomics. 2023 Jul 25;24:419. doi: 10.1186/s12864-023-09441-y (PMC10367311; doi:10.1186/s12864-023-09441-y)
Supplement: Supplementary file 2 — Supplementary Material 2: Fig S2. Sankey diagram of the ceRNA network in CAVD [file 12864_2023_9441_MOESM2_ESM.pdf]

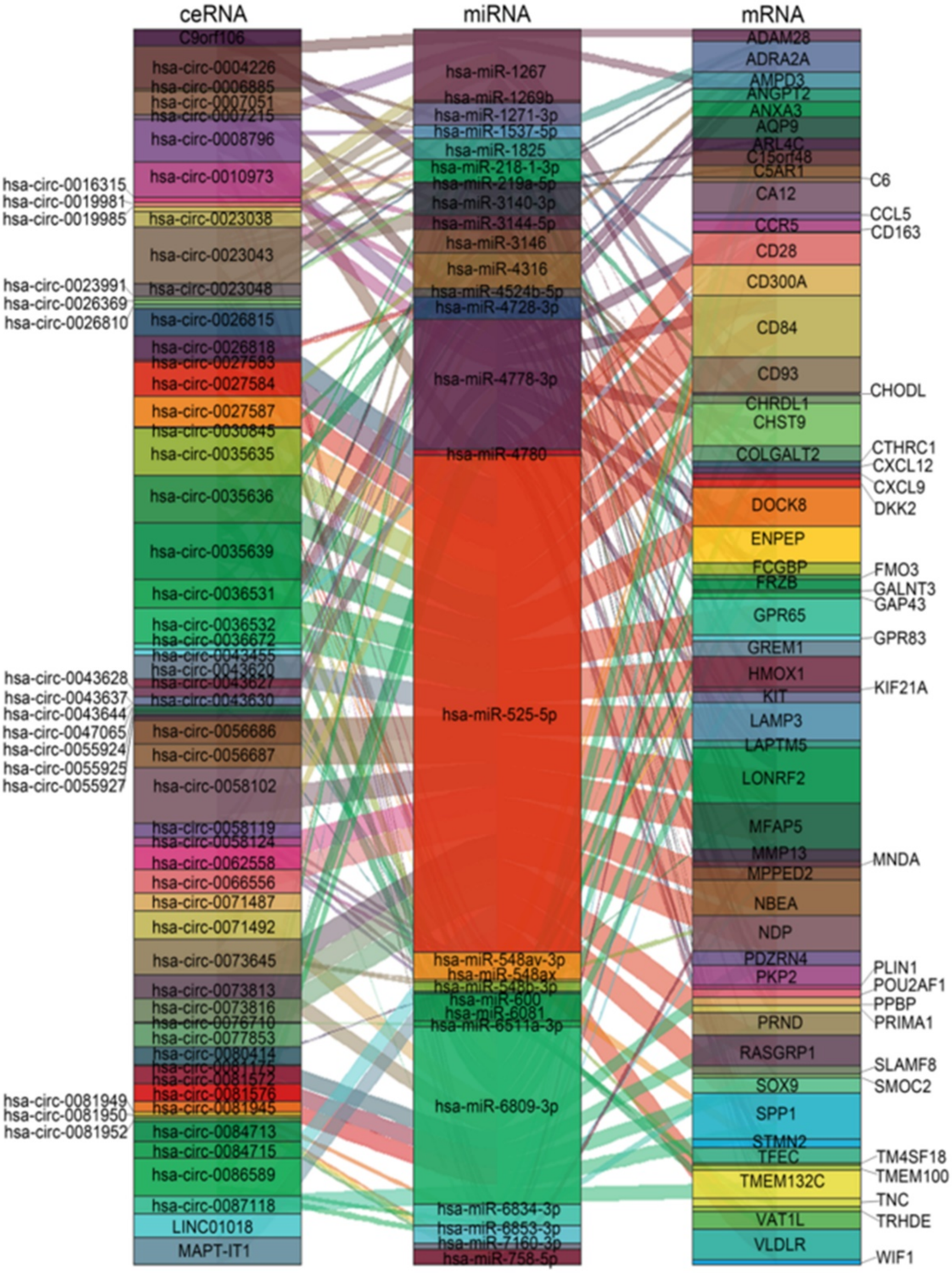

FIGURE S2 | Sankey diagram of the ceRNA network in CAVD. The squareness represents ceRNAs (circRNAs and lncRNAs), miRNAs and mRNAs, the size indicates their degree of connection.
