# Supplementary material for: CircRNA/lncRNA–miRNA–mRNA network and gene landscape in calcific aortic valve disease
Source: BMC Genomics. 2023 Jul 25;24:419. doi: 10.1186/s12864-023-09441-y (PMC10367311; doi:10.1186/s12864-023-09441-y)
Supplement: Supplementary file 3 — Supplementary Material 3: Fig S3. Functional enrichment, pathway analysis and the PPI network construction [file 12864_2023_9441_MOESM3_ESM.pdf]

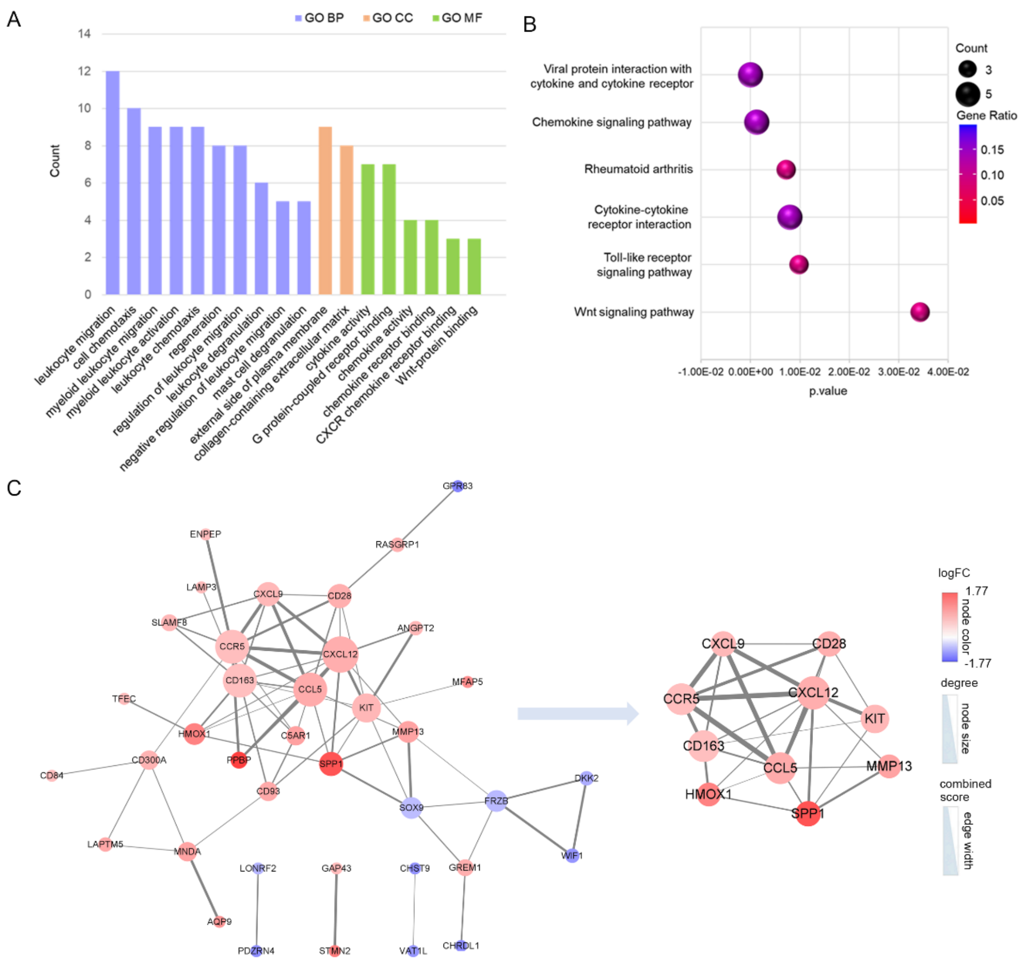

FIGURE S3 | Functional enrichment, pathway analysis and the PPI network construction. (A) The major GO terms of BP, CC and MF. (B) KEGG pathway enrichment. (C) The PPI network of the DEmRNAs in the ceRNA network and the top 10 points in PPI network were selected as hub genes.
