# Supplementary material for: CircRNA/lncRNA–miRNA–mRNA network and gene landscape in calcific aortic valve disease
Source: BMC Genomics. 2023 Jul 25;24:419. doi: 10.1186/s12864-023-09441-y (PMC10367311; doi:10.1186/s12864-023-09441-y)
Supplement: Supplementary file 4 — Supplementary Material 4: Fig S4. Immune cell patterns in CAVD [file 12864_2023_9441_MOESM4_ESM.pdf]

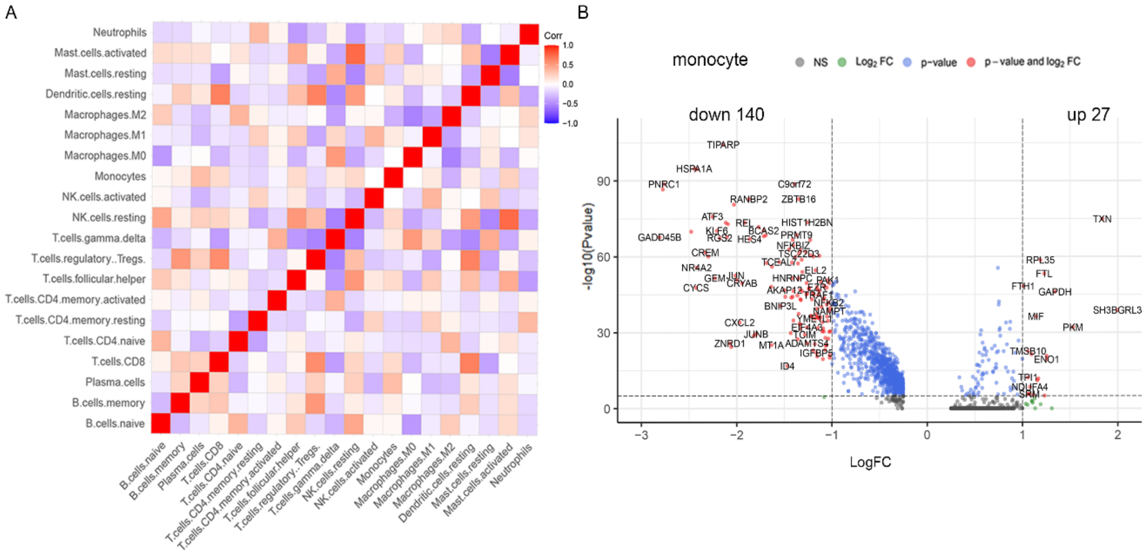

FIGURE S4 | Immune Cell Patterns in CAVD. (A) The correlation analysis between immune cells. (B) Volcano plot of DEGs in monocytes between CAVD and health controls from PRJNA562645.
