# Supplementary material for: CircRNA/lncRNA–miRNA–mRNA network and gene landscape in calcific aortic valve disease
Source: BMC Genomics. 2023 Jul 25;24:419. doi: 10.1186/s12864-023-09441-y (PMC10367311; doi:10.1186/s12864-023-09441-y)
Supplement: Supplementary file 5 — Supplementary Material 5: Fig S5. Identification of CAVD-related DEGs between BAVs and TAVs, and different genders [file 12864_2023_9441_MOESM5_ESM.pdf]

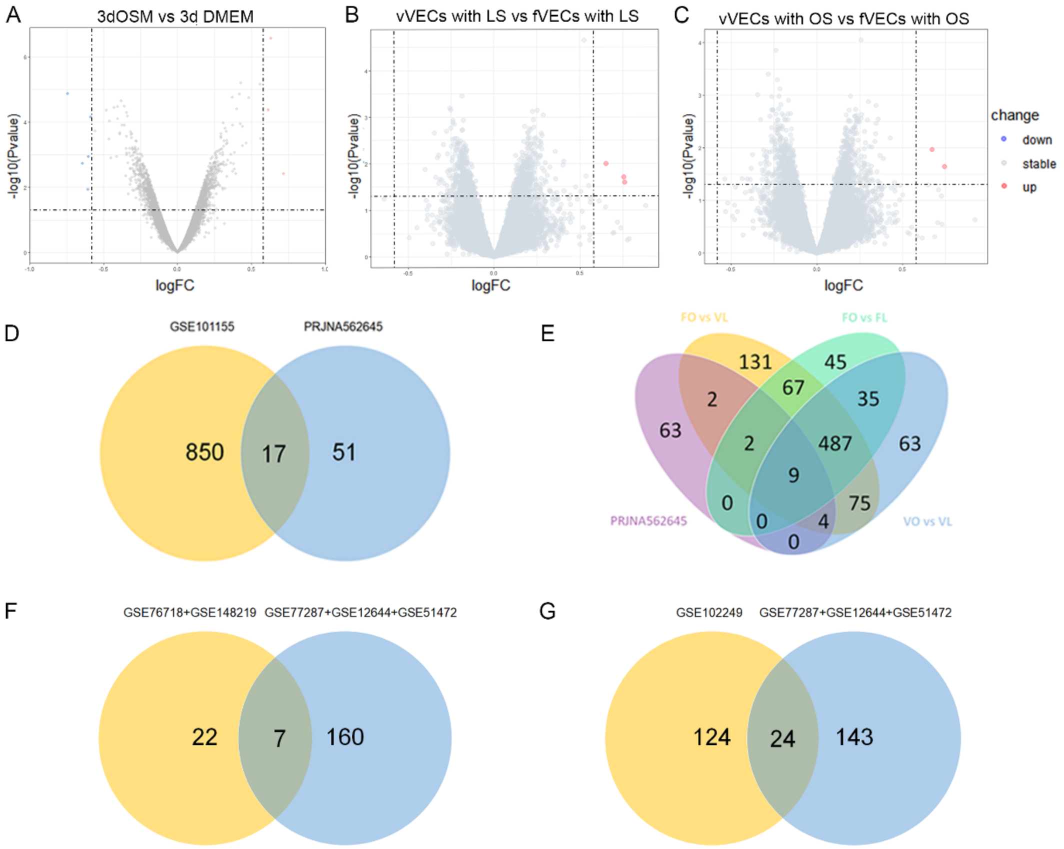

FIGURE S5 | Identification of CAVD-related DEGs between BAVs and TAVs, and different genders. (A) Volcano plot of the DEGs between VICs cultured with OSM or DMEN for 3 days. (B-C) Volcano plot of the DEGs between fVECs and vVECs with LS or OS. (D) Venn diagram of DEGs in VICs from GSE101155 and PRJNA562645. (E) Venn diagram of DEGs in VECs from GSE101155 and PRJNA562645. (F) Venn diagram of DEGs between cBAVs and cTAVs intersection with CAVD-related DEGs from GSE77287, GSE12644 and GSE51472. (G) Venn diagram of DEGs between genders intersection with CAVD-related DEGs.
